# Effect of spinal immobilisation devices on radiation exposure in conventional radiography and computed tomography

**DOI:** 10.1007/s10140-015-1371-0

**Published:** 2016-01-11

**Authors:** Baukje Hemmes, Cécile R. L. P. N. Jeukens, Gerrit J. Kemerink, Peter R.G. Brink, Martijn Poeze

**Affiliations:** Network Acute Care Limburg, Maastricht University Medical Center, Maastricht, The Netherlands; Department of Radiology and Nuclear Medicine, Maastricht University Medical Center, Maastricht, The Netherlands; Department of Surgery, Maastricht University Medical Center, Maastricht, The Netherlands; NUTRIM, School of Nutrition and Translational Research in Metabolism, Maastricht University, Maastricht, The Netherlands

**Keywords:** Spinal immobilisation, Radiation exposure, Image quality, Conventional radiography (CR), Computed tomography (CT)

## Abstract

Trauma patients at risk for, or suspected of, spinal injury are frequently transported to hospital using full spinal immobilisation. At the emergency department, immobilisation is often maintained until radiological work-up is completed. In this study, we examined how these devices influence radiation exposure and noise, as a proxy for objective image quality. Conventional radiographs (CR) and computer tomography (CT) scans were made using a phantom immobilised on two types of spineboard and a vacuum mattress and using two types of headblocks. Images were compared for radiation transmission and quantitative image noise. In CR, up to 23 % and, in CT, up to 11 % of radiation were blocked by the devices. Without compensation for the decreased transmission, noise increased by up to 16 % in CT, depending on the device used. Removing the headblocks led to a statistically significant improvement in transmission with automatic exposure control (AEC) enabled. Physicians should make an informed decision whether the increased radiation exposure outweighs the risk of missing a clinically significant injury by not making a CR or CT scan. Manufacturers of immobilisation devices should take radiological properties of their devices into account in the development and production process.

## Introduction

The spineboard was originally introduced as a means of extricating patients from a crashed vehicle or from a hard-to-reach location [1, 2]. Over the decades, spinal immobilisation using a spineboard or vacuum mattress with a head immobiliser has become the gold standard for prehospital care for trauma patients, including transport of patients on the spineboard into the hospital [3–6]. Although physicians are advised to remove patients from these devices as soon as possible [3–5], many patients undergo primary clinical and radiological evaluation at the emergency department and/or radiology department to rapidly assess life-threatening injuries with the devices in place [7, 8]. Furthermore, whole-body computed tomography (CT) has recently come to be used as a primary diagnostic investigation at the emergency department. Secondary survey frequently includes conventional radiography (CR) and CT scans of the extremities if deemed necessary. This approach is advocated in order to minimise the risk of missing a clinically important injury or unstable spinal fractures that can potentially lead to serious impairment [9, 10] and legal ramifications [11, 12].

Since high image quality of CT scans is a prerequisite for diagnostic workup, any disturbances, such as artefacts or noise, should be kept to a minimum. Artefacts can be limited by avoiding or removing objects with sharp transitions in material density [13–16], while noise reduction can be achieved by increasing the radiation dose. However, exposure to ionising radiation is associated with increased cancer risk [17–20]. Several studies have indicated that not only CT, but, to a lesser degree, also diagnostic conventional radiography (computed radiography (CR)) may account for a non-negligible increased risk of cancer [17, 19, 21]. It is therefore important that the benefits of (increased) radiation exposure should be weighed critically against its risks. In accordance with the as low as reasonably achievable (ALARA) principle in radiography [22], the materials placed between the X-ray source and X-ray detector should have a low level of absorption in order to minimise the radiation dose that the patient is exposed to. In trauma workup, the level of radiation absorption by the spinal immobilisation device may vary significantly [23], thereby altering the radiation dose necessary to deliver high-quality images. In this study, we compared devices for spinal stabilisation in terms of radiation transmission and the effect on objective image quality. The hypothesis in this study was that the devices would differ in the radiation dose necessary for good-quality images. Our aim was to find out which of these devices is the preferred choice for spinal stabilisation from a radiation exposure point of view.

## Materials and methods

### Materials and set-up

**Fig. 1:**
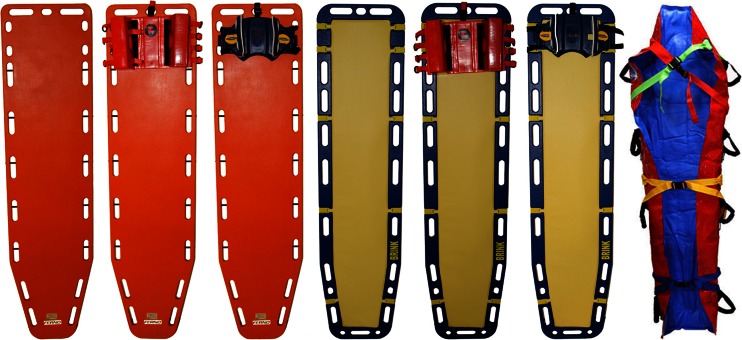
Immobilization devices used in this study. From *left* to *right*: rigid spineboard, no headblocks; rigid spineboard, soft foam headblocks; rigid spineboard, new design headblocks; soft-layered spineboard, no headblocks; soft-layered spineboard, soft foam headblocks; soft-layered spineboard, new design headblocks; vacuum mattress

Radiation transmission and image noise for CR and CT were compared using two spineboards (Millennia Backboard; Ferno-Washington Inc, Wilmington, OH, and a prototype soft-layered long spineboard [24]) in combination with two different headblocks (soft foam headblocks: universal head immobiliser, Ferno-Washington Inc, Wilmington, OH, and new-design headblocks: speedblocks, Laerdal Medical, Stavanger, Norway) or none and a vacuum mattress (RedVac; Radstadt, Austria) (Fig. 1). This means that we measured eight different set-ups (Table 1). The study was approved by the Medical Research Ethics Committee of the Maastricht University Medical Centre and registered as ISRCTN 68626238.

**Table 1:** Set-ups used

Spinal immobilisation	Head immobilisation	CR	CT
None	None	X	X
Rigid	None	X	X
Rigid	Soft foam		X
Rigid	New design		X
Soft layered	None	X	X
Soft layered	Soft foam		X
Soft layered	New design		X
Vacuum mattress	None	X	X

CR images were obtained using an over-Table Philips Trauma Diagnost (Philips, Best, The Netherlands). Transmitted radiation was measured using a 30-mL ionisation chamber and a Capintec 192A electrometer (Capintec, Inc, Ramsey, NJ, USA). A 3-mm-thick lead shield with a 76 × 45-mm window was placed between the device and the ionisation chamber to eliminate scattered radiation as much as possible. The set-up was rebuilt after each image had been taken, to mimic the variation in practical use of the devices. Each set-up was measured five times and with four different technique settings of the equipment, not using the automatic exposure control (AEC) (Table 2).

**Table 2:** Settings used

Setting	Tube potential (kV)	Exposure time (ms)	Tube current time (mAs)
CR setting 1	60	165	100
CR setting 2	75	157	100
CR setting 3	100	220	100
CR setting 4	120	271	100
CT settings (axial head scan, single rotation; collimation 64 × 0.9 mm)	120	n.a.	179

*n.a.* not applicable

CT images were obtained using a dual-source 128 slice CT scanner (Definition Flash, Siemens Healthcare, Forchheim, Germany). A standard 16-cm-diameter computed tomography dose index (CTDI) head phantom was used in combination with a pencil ionisation chamber (Unfors RaySafe; Billdal, Sweden). The ionisation chamber was consecutively inserted in each of the five available holes of the phantom, with the other holes filled up with rods made of the same material as the phantom. The CTDI was subsequently calculated as (1/3 ∗ radiation dose measurement of central hole) + (2/3 ∗ average of the radiation dose measurements of peripheral holes) [25]. The phantom was placed on each of the devices and secured in place using either headblocks or foam spacers (for the spineboards) or by modelling the vacuum mattress around the phantom. This procedure was repeated five times per set-up, thus mimicking variations in realistic placement of both the device and the head.

### Calculations

Transmission, i.e. the percentage of radiation that passed through the device, was calculated as the radiation dose passing with the device in place, divided by the radiation dose without the device in place. For CR, the measured air kerma was taken as the radiation dose, while the CTDI value was used for CT.

**Fig. 2:**
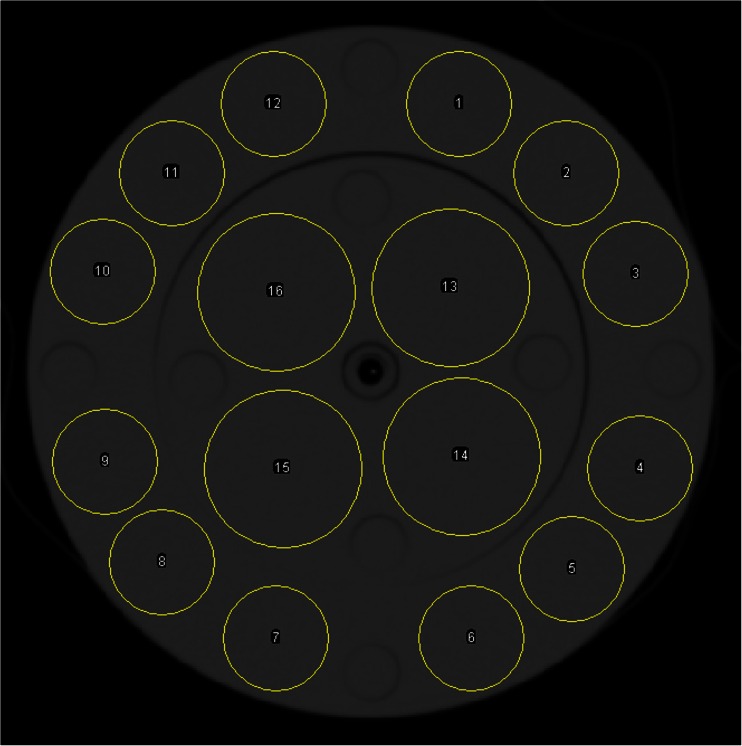
Regions of interest for noise measurements in CT

Image noise in CT was used as a measure of objective image quality. It was defined as the standard deviation (SD) of the CT values (in Hounsfield units) of the pixels within 16 circular regions of interest covering 51 % of the phantom (Fig. 2). Image noise was determined for the middle slice of all phantom scans using ImageJ version 1.47v for Windows software (http://imagej.nih.gov/ij/).

### Statistical analysis

Statistical analyses were performed using SPSS (IBM, Chicago, IL), version 20.0.0. Data are presented as mean ± SD. Non-parametric data were compared using Kruskal–Wallis testing for overall differences and Mann–Whitney *U* test for differences between devices. Parametric data were compared using repeated measure ANOVA for differences between devices. Significance was assumed at *P* < 0.05.

## Results

**Fig. 3:**
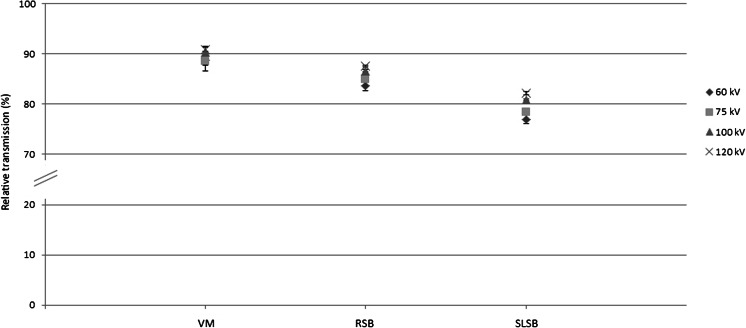
Relative transmission in CR for various devices and different tube settings, relative to no device. Presented data are means ± SD. *VM* vacuum mattress, *RSB* rigid spineboard, *SLSB* soft-layered spineboard

**Fig. 4:**
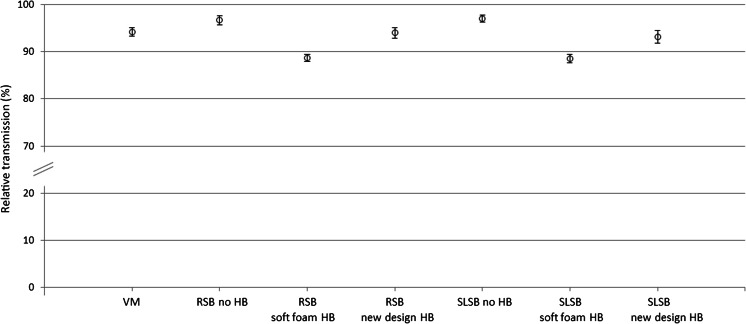
Relative transmission in CT for various devices, relative to no device. Presented data are means ± SD. *VM* vacuum mattress, *RSB* rigid spineboard, *SLSB* soft-layered spineboard, *HB* headblocks. - Significant difference (*P* < 0.05) for no device vs. vacuum mattress, *RSB* new design headblocks, rigid spineboard, *SLSB* new design headblocks, *RSB* soft foam headblocks, *SLSB* soft foam-layered spineboard, *HB* headblocks. - Significant difference (*P* < 0.05) for vacuum mattress vs. SLSB soft foam headblocks. - Significant difference (*P* < 0.05) for SLSB no headblocks vs. SLSB soft foam headblocks

In CR, there was a significant main effect of device on transmission, *F*(2, 8) = 163,914 (*P* < 0.01) and a main effect of voltage on transmission, *F*(3, 12) = 13,030 (*P* < 0.01) (Fig. 3). Subsequent contrast tests revealed that transmission was highest for the vacuum mattress, followed by the rigid spineboard, and lowest for the soft-layered spineboard. These results were seen at all voltages. A significant interaction effect indicated that the differences between the devices became more pronounced with increasing voltages, *F*(6, 24) = 4787 (*P* < 0.01). In CT, transmission was significantly lower when headblocks were used compared to no headblocks (Fig. 4). Kruskal–Wallis testing for non-parametric groups showed an overall significant difference between the conditions (*P* < 0.001).

For all tube voltages, Mann–Whitney tests showed significant differences after Bonferroni correction between all three devices at *P* < 0.0083

**Fig. 5:**
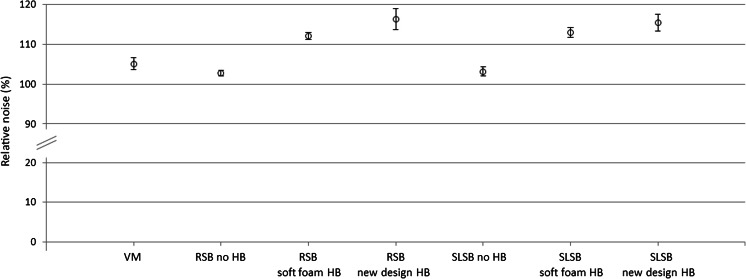
Relative noise in CT for various devices, relative to no device. Presented data are means ± SD. *VM* vacuum mattress, *RSB* rigid spineboard, *SLSB* soft-layered spineboard, *HB* headblocks. - Significant difference** (*P* < 0.05) for no device vs. RSB new design headblocks, SLSB no headblocks, SLSB soft foam headblocks and SLSB new design headblocks. - Significant difference** (*P* < 0.05) for vacuum mattress vs. RSB soft foam headblocks, RSB new design headblocks, SLSB soft foam headblocks and SLSB new design headblocks. - Significant difference** (*P* < 0.05) for RSB no headblocks vs. RSB soft foam headblocks, RSB new design headblocks, SLSB soft foam headblocks and SLSB new design headblocks. - Significant difference** (*P* < 0.05) for RSB soft foam headblocks vs. RSB new design headblocks, SLSB no headblocks and SLSB new design headblocks. - Significant difference** (*P* < 0.05) for RSB new design headblocks vs. SLSB no headblocks and SLSB soft foam headblocks. - Significant difference** (*P* < 0.05) for SLSB no headblocks vs. SLSB soft foam headblocks and SLSB new design headblocks. **Significant differences after Bonferroni correction

With regard to noise in CT, repeated measures ANOVA showed significant differences in noise between all conditions, *F*(7, 14) = 60.9, *P* < 0.01 (Fig. 5). As expected, a significant relationship was found between decreased transmission and increased noise in CT, *r* = 0.67, *P* < 0.01.

## Discussion

This study compared a number of devices for spinal immobilisation with regard to radiation transmission and objective image quality. Although adding a spineboard or vacuum mattress to the set-up decreased both transmission and image quality, adding headblocks to the spineboards decreased transmission and image quality even more.

Despite radiation exposure in acute (trauma) care being an important issue [26–29], studies examining radiation transmission in spineboards and vacuum mattresses have been sparse [23]. Our study adds to this knowledge of the radiographic properties of spineboards and vacuum mattresses, by including data regarding headblocks, which are considered an integral part of the equipment used to achieve full spinal immobilisation [30]. Current trauma protocols require the use of these devices, which absorb part of the radiation. CR and CT machines automatically adjust radiation dose to compensate for image quality loss due to absorption, but since any increase in radiation exposure creates an additional cancer risk [20, 21, 31, 32], it is important that the devices have high transmission rates.

Our results show that in CR, transmission decreased by 9 to 23 %, depending on the device and tube voltage used. In CT, the transmission decreased by up to 11 %. When X-rays encounter any form of matter, they are partly transmitted and partly absorbed and scattered. The resulting transmission depends on the energy-dependent attenuation coefficient of the material and the X-ray energy (here determined by the system’s tube voltage). The attenuation coefficient increases when the X-ray energy decreases. Consequently, it is to be expected that the transmission will be reduced when the tube voltage is lowered, as we have shown in Fig. 3. The observed differences in transmission between the set-ups are related to differences in material properties (i.e. attenuation coefficients) as well as differences in the amount of material present (compare the set-up with and without headblocks present).

For all devices, transmission was higher for CT than for CR. This can be explained by considering that, in contrast to CR, only some of the projections acquired in a CT scan involve X-ray trajectories through the device. In addition, the use of a thicker beam filter in CT results in more penetrating radiation compared to CR, even at the same tube voltage. As trauma CRs are usually taken in anterior–posterior (AP) direction, the patient is actually being exposed to additional radiation dose, because the posteriorly placed immobilisation device absorbs part of the radiation that has already passed through the patient. However, it should be noted that although the relative extra radiation exposure is higher for CR than CT, the absolute actual dose increase is in the order of five times higher for CT, as the CT radiation exposure is higher to start with. Regardless of this, with ever more institutions moving away from CR and using CT instead [33], radiation protection according to ALARA principle should always be a priority. Our study shows that one simple way to reduce radiation exposure is to remove the headblocks before CT imaging.

Nonetheless, in case of restless or non-responsive trauma patients at risk for spinal injury, physicians are reluctant to remove the immobilisation devices before imaging has been completed, for fear of worsening or missing an injury [34] and possible consequent medical litigation [12]. However, no studies have so far been published showing a direct causal link between the use of immobilisation devices (or lack thereof) and injury outcome [35]. There are, however, sound arguments to permit removal of the devices in calm, cooperative patients [36].

Since increased exposure to ionisation radiation induces health risks [31, 32], even though the risks are small and consequently associated with large uncertainties [37], any measure to reduce radiation should be welcomed. A total-body trauma CT scan without immobilisation devices exposes the patient to 15–30 mSv [38–40]. When this scan is performed on a rigid spineboard with soft foam headblocks in place, an increase in radiation exposure of 11 % is needed to maintain image quality, increasing the radiation dose by 1.7–3.4 mSv. Data from earlier studies on the risks of radiation exposure [31, 32] indicate that the additional risk of dying from cancer is 0.5 % per 100 mSv, assuming an estimated overall lifetime cancer risk of 25 %. Although the added risk of fatal cancer due to the use of devices for spinal immobilisation may be small, physicians should make an informed decision not only on whether or not to make use CR or CT in on an immobilised patient, but also on the question whether some of the devices can be removed before imaging, factoring in both the radiation risks and the risks of potentially missed injuries. Furthermore, designers of devices for spinal immobilisation should take into account the radiological properties of their devices.

Some remarks can be made with regard to our study. First, we only looked at noise as an indicator of image quality. We did not look at other factors, such as artefacts (beam hardening, additional scattering and banding) caused by differences in attenuation by the devices. This is becoming increasingly important, as there is a trend towards lower dosages, which in turn may result in more visible artefacts. This subject is discussed in detail elsewhere [13]. Second, we evaluated only one CT scanning protocol using one multidetector CT scanner. We chose to consider only a tube voltage of 120 kV, as this is the clinical standard for trauma diagnostics in our hospital. Although beam filtration may be different for CT scanners of other manufacturers, resulting in somewhat different beam qualities, we believe that the results will be qualitatively comparable.

## Conclusion

There are significant differences in radiation transmission depending on the type of immobilisation device used. In CR, the vacuum mattress showed the highest transmission rates, while in CT, the spineboards without headblocks performed best in terms of both transmission and noise. In view of the duty to keep patient radiation exposure as low as possible while still achieving the required image quality, the results of this study can help in making an informed decision whether the risks associated with radiation exposure outweigh those of missed injuries.

## References

[1] Farrington JD (1968). Extrication of victims—surgical principles. J Trauma.

[2] Farrington JD (1967). Death in a ditch. Bull AM Coll Sur.

[3] Cooke MW (1998). Use of the spinal board within the accident and emergency department. J Accid Emerg Med.

[4] Lerner EB, Moscati R (2000). Duration of patient immobilization in the ED. Am J Emerg Med.

[5] Malik MHA, Lovell ME (2003). Current spinal board usage in emergency departments across the UK. Injury.

[6] Stagg MJ, Lovell ME (2008). A repeat audit of spinal board usage in the emergency department. Injury.

[7] El-Khoury GY, Kathol MH, Daniel WW (1995). Imaging of acute injuries of the cervical spine: value of plain radiography, CT, and MR imaging. Am J Roentgenol.

[8] France JC, Bono CM, Vaccaro AR (2005). Initial radiographic evaluation of the spine after trauma: when, what, where, and how to image the acutely traumatized spine. J Orthop Trauma.

[9] Hadida C, Lamire J (1997). Missed upper cervical spine fracture: clinical and radiological considerations. J Can Chiropr Assoc.

[10] Meek S (1998). Fractures of the thoracolumbar spine in major trauma patients. BMJ: Br Med J.

[11] Berlin L (2003). CT versus radiography for initial evaluation of cervical spine trauma: what is the standard of care?. Am J Roentgenol.

[12] Lekovic GP (2007). Litigation of missed cervical spine injuries in patients presenting with blunt traumatic injury. Neurosurgery.

[13] Hemmes B, Jeukens CRLPN, Al-Haidaari A, et al. Effect of spineboard and headblocks on the image quality of head CT scans. Submitted 10.1007/s10140-016-1396-z PMC4875944 27091739

[14] Miller JA, Mele C, Abu-Judeh H (1999). Significance of backboard artifacts on portable trauma series chest radiographs. Emerg Radiol.

[15] Daffner RH, Khoury MB (1987). Pseudofractures due to Nec-Loc cervical immobilization collar. Skeletal Radiol.

[16] Schou J, Kiermayer H, Ummenhofer W, Herion H-P (2001). In search of the most suitable technique for truncal spinal immobilization with associated radiography. Eur J Emerg Med.

[17] Berrington de Gonzalez A, Darby S (2004). Risk of cancer from diagnostic x-rays: estimates for the UK and 14 other countries. Lancet.

[18] Shimizu Y, Schull WJ, Kato H (1990). Cancer risk among atomic bomb survivors. RERF Life Span Study JAMA.

[19] Einstein AJ, Henzlova MJ, Rajagopalan S (2007). Estimating risk of cancer associated with radiation exposure from 64-slice computed tomography coronary angiography. JAMA.

[20] Hall EJ, Brenner DJ (2008). Cancer risks from diagnostic radiology. Br J Radiol.

[21] Levy AR, Goldberg MS, Hanley JA, Mayo NE, Benoit P (1994). Projecting the lifetime risk of cancer from exposure to diagnostic ionising radiation for adolescent idiopathic scoliosis. Health Phys.

[22] International Commission on Radiological Protection (1977) Radiation protection. Annals of the ICRP, Publication 26 (New York: Pergamon Press)

[23] Linsenmaier U, Krotz M, Kanz K-G (2001). Evaluation von Wirbelsaulen-brettern fur die Roentgendiagnostik. Fortschr Rontgenstr.

[24] Hemmes B, Poeze M, Brink PRG (2010). Reduced tissue-interface pressure and increased comfort on a newly developed soft-layered long spineboard. J Trauma.

[25] Menzel H, Schibilla H, Teunen D (2000). European guidelines on quality criteria for computed tomography.

[26] Bregstein JS, Lubell TR, Ruscica AM, Roskind CG (2014). Nuking the radiation: minimizing radiation exposure in the evaluation of pediatric blunt trauma. Curr Opin Pediatr.

[27] Kruger JF, Chen AH, Rybkin A, Leeds K, Frosch DL & Goldman LE (2014) Clinician perspectives on considering radiation exposure to patients when ordering imaging tests: a qualitative study. BMJ Qual Saf 10.1136/bmjqs-2013-002773 24764135

[28] Corwin MT, Sheen L, Kuramoto A, Lamba R, Parthasarathy S, Holmes JF (2014). Utilization of a clinical prediction rule for abdominal-pelvic CT scans in patients with blunt abdominal trauma. Emerg Radiol.

[29] Mills AM, Raja AS, Marin JR (2015). Optimizing diagnostic imaging in the emergency department. Acad Emerg Med.

[30] American College of Surgeons, ed. (2012) Advanced trauma life support. Student course manual, 9th ed. Chicago, IL

[31] International Commission on Radiological Protection (2007). The 2007 recommendations of the International Commission on Radiological Protection. Ann ICRP.

[32] National Research Council, Health risks from exposure to low levels of ionizing radiation: BEIR VII Phase 2 (2006), Washington DC: National Academic Press 25077203

[33] Heller MT, Kanal E, Almusa O (2014). Utility of additional CT examinations driven by completion of a standard trauma imaging protocol in patients transferred for minor trauma. Emerge.

[34] Morrissey JF, Kusel ER, Sporer KA (2014). Spinal motion restriction: an educational and implementation program to redefine prehospital spinal assessment and care. Prehosp Emerg Care.

[35] Kwan I, Bunn F & Roberts IG (2009) Spinal immobilisation for trauma patients (review). Cochrane database of systematic reviews 10.1002/14651858.CD002803 PMC7003994 11406043

[36] Hauswald M (2013). A re-conceptualisation of acute spinal care. Emerg Med J.

[37] Hendee WR, O'Connor MK (2012). Radiation risks of medical imaging: separating fact from fantasy. Radiology.

[38] Brenner DJ, Doll R, Goodhead DT (2003). Cancer risk attributable to low doses of ionizing radiation: assessing what we already know. Proc Natl Acad Sci U S A.

[39] Sierink JC, Saltzherr TP, Wirtz MR, Streekstra GJ, Beenen LFM, Goslings JC (2013). Radiation exposure before and after the introduction of a dedicated total-body CT protocol in multitrauma patients. Emerg Radiol.

[40] Mettler FA, Huda W, Yoshizumi TT, Mahesh M (2008). Effective doses in radiology and diagnostic nuclear medicine: a catalog. Radiology.

